# Comparative Efficacy of Three Commercial PCV2 and *Mycoplasma hyopneumoniae* Bivalent Vaccines Against Experimental *M. hyopneumoniae* Challenge

**DOI:** 10.3390/vaccines14100853

**Published:** 2026-09-28

**Authors:** Yuan Gan, Lei Zhang, Yanna Wei, Istvan Kiss, Philippe Mazerolles, Renxuan Wang, Ting Yuan, Jia Wang, Hao Wang, Yongxu Zhao

**Affiliations:** 1 Key Laboratory of Veterinary Biological Engineering and Technology, Institute of Veterinary Medicine, Jiangsu Academy of Agricultural Sciences, Ministry of Agriculture and Rural Affairs, Nanjing 210014, China; 2 Guotai (Taizhou) Center of Technology Innovation for Veterinary Biologicals, Taizhou 225300, China; 3 College of Veterinary Medicine, Nanjing Agricultural University, Nanjing 210095, China; 4 Ceva Animal Health, 33500 Libourne, France; istvan.kiss@ceva.com (I.K.);

**Keywords:** *Mycoplasma hyopneumoniae*, porcine circovirus type 2, PCV2-Mhp bivalent vaccine, Cirbloc^®^ M Hyo, experimental challenge, enzootic pneumonia

## Abstract

**Background/Objectives:** *Mycoplasma hyopneumoniae* (Mhp), porcine circovirus type 2 (PCV2) and porcine reproductive and respiratory syndrome virus (PRRSV) are major contributors to porcine respiratory disease complex (PRDC), causing substantial economic losses. **Methods:** This study evaluated a commercial PCV2-Mhp bivalent vaccine, Cirbloc^®^ M Hyo (Vaccine A), containing recombinant PCV2 Cap protein and inactivated Mhp antigen, vs. two reference bivalent vaccines (B and C) in an Mhp challenge model. Eighty-two healthy 3-week-old specific-pathogen-free piglets were randomly allocated to five groups (three vaccinated, unvaccinated challenged control, unvaccinated unchallenged control). Pigs received a single intramuscular vaccination at 3 weeks of age and were challenged intratracheally with virulent Mhp lung homogenate five weeks later. Clinical signs, serology, lung lesions, histopathology, and Mhp DNA detection in lungs were assessed. **Results:** No vaccine-related adverse reactions occurred. Vaccine A induced earlier and higher Mhp-IgG seroconversion before challenge than reference vaccines. Following challenge, Vaccine A significantly reduced mean lung lesion scores compared with the unvaccinated challenged control (2.28 ± 2.37 vs. 6.56 ± 4.03; *p* < 0.05), with markedly reduced peribronchiolar inflammation and lymphoid hyperplasia and the lowest Mhp DNA detection rate among challenged groups. **Conclusions:** Cirbloc^®^ M Hyo provided enhanced protection against experimental Mhp challenge, as shown by reduced lung lesions and milder histopathological changes, supporting its use for comprehensive PRDC management.

## 1. Introduction

Porcine respiratory disease complex (PRDC) is a multifactorial syndrome that severely affects the health and productivity of growing-finishing pigs worldwide. It results from complex interactions among viral and bacterial pathogens, environmental stressors, management practices, and host immunological factors [1,2]. Among the major pathogens involved in PRDC, *Mycoplasma hyopneumoniae* (Mhp), the causative agent of enzootic pneumonia (EP), porcine circovirus type 2 (PCV2) and porcine reproductive and respiratory syndrome virus (PRRSV) are recognized as three of the most economically important agents in modern swine production [3]. Mhp primarily colonizes the ciliated epithelium of the respiratory tract, disrupting mucociliary clearance and predisposing pigs to secondary bacterial infections. PCV2, in contrast, is associated with lymphoid depletion and immune dysregulation, which may further aggravate polymicrobial respiratory disease and reduce overall herd performance [4,5].

Vaccination remains one of the most important tools for controlling both Mhp- and PCV2-associated diseases. For Mhp, vaccination is mainly intended to reduce clinical respiratory signs, decrease the severity of pulmonary lesions, and improve production performance under field conditions [3,6]. Ready-to-use single-dose PCV2-Mhp combination vaccines allow both antigens to be administered in a single injection, simplifying vaccination programs [7,8,9].

However, the protective efficacy of commercial PCV2-Mhp combination vaccines against Mhp may vary among products and experimental conditions. Current Mhp bacterin-based vaccines generally provide partial protection by reducing clinical signs and lung lesion severity, but they do not always prevent infection or pathogen colonization [7,10,11]. Given the continued global importance of Mhp and its role in polymicrobial PRDC, controlled experimental challenge studies remain essential for evaluating and comparing vaccine-induced protection. Experimental challenge studies have demonstrated that commercial Mhp vaccines and PCV2-Mhp combination vaccines can reduce Mhp-associated lung lesions following challenge [7,8,9,12].

The present study was designed to evaluate the efficacy of Cirbloc^®^ M Hyo, a commercial PCV2-Mhp bivalent vaccine, in comparison with two commercial PCV2-Mhp bivalent reference vaccines (Vaccine B and Vaccine C). Given the potential variation in protection against Mhp among commercial bivalent vaccines, an Mhp-only challenge model was used, with the primary objective of comparing vaccine-induced protection against Mhp-associated pulmonary lesions. PCV2-specific antibody responses were assessed as an additional measure of vaccine immunogenicity.

## 2. Materials and Methods

### 2.1. Animals and Housing

A total of 82 healthy 19-day-old crossbred piglets (Landrace × Yorkshire × Duroc) were obtained from a specific-pathogen-free commercial herd confirmed negative for *Mycoplasma hyopneumoniae* (Mhp), porcine circovirus type 2 (PCV2), porcine reproductive and respiratory syndrome virus (PRRSV), African swine fever virus (ASFV), pseudorabies virus (PRV), and swine influenza virus (SIV) by commercial ELISA and quantitative PCR assays. Before enrollment, all piglets were seronegative for Mhp-specific IgG and PCV2-specific IgM/IgG, as determined using commercial kits (IDvet, Grabels, France; Ingezim, Madrid, Spain).

Piglets were housed in isolated, climate-controlled rooms maintained at 22–26 °C and 50–60% relative humidity, with ad libitum access to antibiotic-free creep feed (ADM, Type 086) and drinking water. After a 2-day acclimatization period (Days −2 to 0), piglets were stratified by body weight and sex and randomly assigned to five groups using a computer-generated randomization table in IBM SPSS Statistics 26.0 (IBM Corp., Armonk, NY, USA).

### 2.2. Vaccines and Experimental Design

Three commercial PCV2-Mhp bivalent vaccines, designated Vaccines A–C, were stored and administered according to the manufacturers’ instructions. Vaccine composition and experimental design are summarized in Table 1. On Day 0, at 3 weeks of age, pigs in the vaccinated groups received a single 2.0 mL intramuscular injection of the assigned vaccine in the left cervical triangular muscle. Pigs in the UnVac/Ch and UnVac/UnCh control groups received an equal volume of sterile phosphate-buffered saline (PBS; 2.0 mL, pH 7.4) via the same route.

### 2.3. Challenge Procedure

On Day 35, 5 weeks after vaccination, pigs in the three vaccinated groups and the UnVac/Ch group were challenged intratracheally with 5.0 mL of a virulent, tissue-derived Mhp pneumonic lung homogenate (Batch Mhp20240221) containing 2 × 10^6^ color change units of Mhp. The challenge material was provided by the Institute of Veterinary Medicine, Jiangsu Academy of Agricultural Sciences, propagated in KM2 medium at 37 °C for 72 h, and titrated using the color change unit method. The UnVac/UnCh group remained unchallenged.

### 2.4. Clinical Observations and Performance

Pigs were monitored daily for 7 days after vaccination for local injection-site reactions, including redness, swelling, and induration > 2 cm, as well as systemic clinical signs such as appetite, behavior, and respiratory status. From Day 35, the day of challenge, to Day 63, the day of necropsy, coughing and respiratory signs were assessed twice daily at 10:00 a.m. and 4:00 p.m.

Body weight was recorded on Day-2 before vaccination, Day 35 before challenge, and Day 63 at necropsy. Average daily weight gain (ADWG) was calculated for the entire study period from Day-2 to Day 63 and for the post-challenge period from Day 35 to Day 63.

### 2.5. Serology

Blood samples were collected on Days -2 before vaccination, 21, 35, 49, and 63. Mhp-specific serum IgG antibodies were detected using a commercial ELISA kit (ID Screen^®^ *Mycoplasma hyopneumoniae* Competition ELISA kit; IDvet, Grabels, France). PCV2-specific IgG and IgM antibodies were assessed using commercial ELISA kits (Ingezim Circo IgG/IgM; 11.PCV.K2/11.PCV.K1; Ingenasa, Madrid, Spain). All assays were performed according to the manufacturers’ instructions.

### 2.6. Necropsy and Lung Lesion Scoring

On Day 63, corresponding to 4 weeks post-challenge, all pigs were humanely euthanized and subjected to necropsy. Macroscopic lung lesions compatible with enzootic pneumonia (EP) were evaluated by a blinded veterinary pathologist using a 28-point scoring system [13].

### 2.7. Histopathological Examination

Representative lung tissue samples were collected from the junction between affected and grossly normal parenchyma. For pigs without visible lung lesions, samples were collected from corresponding anatomical regions. Tissue samples were fixed in 10% neutral buffered formalin, embedded in paraffin, sectioned at 4 μm, and stained with hematoxylin and eosin (H & E). Slides were examined under an Olympus BX53 light microscope (Olympus Corporation, Tokyo, Japan) for characteristic EP-associated lesions, including peribronchiolar lymphoid hyperplasia, inflammatory cell infiltration, and alveolar exudation.

### 2.8. Mhp DNA Quantification in Lung Tissue

Mhp DNA in lung tissue homogenates was quantified using a TaqMan-based real-time qPCR assay targeting the Mhp P97 gene. The following primers and probe were used: forward primer 5′-CC AGA ACC AAA TTC CTT CGC TG-3′; reverse primer 5′-ACT GGC TGA ACT TCA TCT GGG CTA-3′; and probe 5′-FAM-AGC AGA TCT TAG TCA AAG TGC CCG TG-BHQ-3′.

The assay was performed using a StepOnePlus Real-Time PCR System (Thermo Fisher Scientific, Waltham, MA, USA). Absolute quantification was performed using a standard curve generated from serial 10-fold dilutions of a recombinant plasmid containing the Mhp P97 target sequence. Mhp DNA loads were expressed as DNA copies per gram of lung tissue (copies/g). The detection limit of the assay was 10 copies/μL of plasmid standard.

### 2.9. Exclusion of Co-Infections

To rule out interference from other major swine respiratory pathogens, serum and tissue samples were tested using a commercial ASFV qPCR kit (IDEXX Laboratories, Inc., Westbrook, ME, USA), PRV qPCR kit (Wuhan Keqian Biology Co., Ltd., Wuhan, China), PRRSV and SIV RT-qPCR kit (Thermo Fisher Scientific, Waltham, MA, USA), according to the manufacturers’ instructions.

### 2.10. Statistical Analysis

Data were analyzed using GraphPad Prism 8.0.1 (GraphPad Software, San Diego, CA, USA) and SAS 9.4 (SAS Institute Inc., Cary, NC, USA). Data are presented as mean ± standard deviation. Normality was assessed using the Shapiro–Wilk test. Body weight and ADWG met the normality assumption and were analyzed using two-way ANOVA. Lung lesion scores, quantitative antibody measurements, and Mhp DNA quantities were analyzed using the Kruskal–Wallis test followed by Dunn’s test with Bonferroni adjustment. Antibody measurements were compared separately at each sampling time. Mhp DNA quantities were compared among the four challenged groups, with non-detects ranked below detectable values. Seroconversion and Mhp DNA detection rates were compared using two-sided Fisher’s exact tests with Holm adjustment. *p* < 0.05 was considered statistically significant, using adjusted *p* values for multiple comparisons.

## 3. Results

### 3.1. Clinical Observations and Safety

No vaccine-related systemic reactions, including anorexia, lethargy or pyrexia, and no local injection-site lesions, such as swelling, redness or induration, were observed in any vaccinated group during the 7-day post-vaccination monitoring period. These findings indicated that all three vaccines were well tolerated under the conditions of this study. One pig in the Vaccine C group died on Day 35 due to stress, which was considered unrelated to vaccination or Mhp challenge based on necropsy findings. Therefore, 17 pigs in the Vaccine C group were included in the subsequent post-challenge observations and analyses.

Following Mhp challenge, coughing was only sporadically observed across the challenged groups (Table 2). No coughing or respiratory signs were recorded from Day 35 to Day 49. On Day 56, mild coughing was observed in one pig in the Vaccine A group and one pig in the Vaccine B group. On Day 63, coughing was recorded in 1/18 pigs in the Vaccine A group, 1/18 pigs in the Vaccine B group, 1/17 pigs in the Vaccine C group, and 2/18 pigs in the UnVac/Ch group. No coughing or respiratory distress was observed in the UnVac/UnCh group throughout the observation period.

### 3.2. Body Weight and Average Daily Gain (ADWG)

No significant differences in body weight were observed among groups at baseline (Day-2), before challenge (Day 35), or at necropsy (Day 63) (*p* > 0.05; Table 3). Similarly, no statistically significant differences were detected in ADWG among the vaccinated and control groups during the overall study period from Day-2 to Day 63 or during the post-challenge period from Day 35 to Day 63 (*p* > 0.05). These results indicate that Mhp challenge did not result in measurable differences in growth performance under the conditions of this experimental model.

### 3.3. Detection of Mhp-Specific Antibodies

Mhp-specific IgG seroconversion rates are summarized in Table 4, and antibody dynamics are shown in Figure 1. At 21 days post-vaccination (Day 21), seroconversion was observed in 56% of pigs in the Vaccine A group (10/18) and 33% of pigs in the Vaccine B group (6/18), whereas pigs in the Vaccine C, UnVac/Ch, and UnVac/UnCh groups remained seronegative. By Day 35, corresponding to the day of challenge, the seropositivity rate increased to 72% in Vaccine A group (13/18) and 44% in Vaccine B (8/18). No Mhp-IgG seroconversion was detected in the Vaccine C group prior to challenge.

Following Mhp challenge, seroconversion reached 100% in both the Vaccine A and Vaccine B groups by Day 49, corresponding to 14 days post-challenge. In contrast, seroconversion in the Vaccine C and UnVac/Ch groups was only detected at Day 63, with seropositivity rates of 41% and 39%, respectively. Pigs in the UnVac/UnCh group remained seronegative throughout the study. The proportion of seropositive pigs was numerically higher in the Vaccine A group than in the Vaccine B and Vaccine C groups at both pre-challenge sampling points (Days 21 and 35).

### 3.4. Serology for PCV2

All three vaccinated groups developed clear serological responses to PCV2 after vaccination (Table 5). By Day 21, the PCV2 antibody seropositivity rates were 72.22% in the Vaccine A group, 66.67% in the Vaccine B group, and 61.11% in the Vaccine C group. By Day 35, all pigs in the three vaccinated groups were seropositive for PCV2 and remained seropositive until Day 63.

In contrast, pigs in the UnVac/Ch and UnVac/UnCh control groups remained seronegative for PCV2 throughout the study. These results indicate that all three PCV2-Mhp bivalent vaccines induced PCV2-specific antibody responses, while no detectable PCV2 serological exposure occurred in the unvaccinated control groups during the experimental period.

### 3.5. Gross Pathology and Lung Lesion Scores

At necropsy on Day 63, no visible lung lesions were observed in pigs from the UnVac/UnCh control group. In contrast, pigs in the UnVac/Ch group showed typical macroscopic lesions compatible with enzootic pneumonia, characterized by purple-to-red consolidated areas predominantly located in the apical and cardiac lobes. Macroscopic lung lesions were also observed in the vaccinated groups, but their severity and extent varied among vaccines. Pigs vaccinated with Vaccine A generally showed only minimal and focal lung lesions (Figure 2).

Quantitative analysis of macroscopic lung lesion scores supported these gross pathological observations (Table 6 and Figure 3). Lung lesion scores were significantly lower in the Vaccine A group than in the UnVac/Ch group (mean ± SD: 2.28 ± 2.37 vs. 6.56 ± 4.03; Dunn’s adjusted *p* = 0.0160). No statistically significant difference was observed between the Vaccine A group and the UnVac/UnCh group (0.00 ± 0.00). In contrast, the mean lung lesion scores in the Vaccine B group (5.00 ± 4.98) and Vaccine C group (5.47 ± 5.72) were not significantly different from that of the UnVac/Ch group (*p* > 0.05). The numbers of pigs with complete protection against macroscopic lung lesions were 6/18 in the Vaccine A group, 4/18 in the Vaccine B group, and 3/17 in the Vaccine C group. Among the three vaccinated groups, Vaccine A had the numerically lowest mean macroscopic lung lesion score and showed a significant reduction compared with the UnVac/Ch group.

### 3.6. Histopathological Findings

Microscopic examination of lung sections from the UnVac/Ch group revealed typical histopathological changes compatible with Mhp infection, including severe peribronchiolar and perivascular lymphoid hyperplasia, marked inflammatory cell infiltration in the bronchiolar lumen, and alveolar exudation containing sloughed epithelial cells, neutrophils, and mononuclear cells.

In contrast, lung sections from the Vaccine A group showed markedly milder histopathological changes. Histopathological lesions were observed in 9 of 18 pigs in this group and were mainly characterized by mild focal peribronchiolar inflammatory infiltration with minimal lymphoid aggregation. Overall, the lung architecture in the Vaccine A group was largely preserved and resembled that of the UnVac/UnCh group.

In the Vaccine B and Vaccine C groups, histopathological lesions were observed in 15 of 18 pigs and 14 of 17 pigs, respectively. These lesions were generally moderate to severe, including peribronchiolar lymphoid hyperplasia and inflammatory cell infiltration, and were more similar to those observed in the UnVac/Ch group (Figure 4).

### 3.7. Quantification of Mhp DNA in Lung Tissues

Mhp DNA levels in lung tissues were quantified by qPCR and expressed as copies per gram of tissue (copies/g) (Figure 5). Mhp DNA was detected in all challenged groups, whereas no Mhp DNA was detected in the UnVac/UnCh group.

Among the challenged groups, the Vaccine A group showed the lowest Mhp DNA detection rate and a numerically lower Mhp DNA load in lung tissues compared with the Vaccine B, Vaccine C, and UnVac/Ch groups. The Mhp DNA detection rate was 72.22% in the Vaccine A group, compared with 83.33% in the Vaccine B group, 82.35% in the Vaccine C group, and 100.00% in the UnVac/Ch group. However, neither Mhp DNA detection rates nor DNA loads differed significantly among the three vaccinated groups (*p* > 0.05).

## 4. Discussion

Effective control of porcine respiratory disease complex (PRDC) remains an important challenge for global swine production. Combination vaccines targeting major respiratory pathogens such as *Mycoplasma hyopneumoniae* (Mhp) and porcine circovirus type 2 (PCV2) have been widely adopted in commercial pig production because they simplify vaccination programs and reduce animal handling. However, comparative efficacy data generated under controlled experimental challenge conditions remain important for evaluating vaccine performance and supporting evidence-based vaccine selection [7,14].

In the present study, we compared the efficacy of Cirbloc^®^ M Hyo (Vaccine A) with two commercial PCV2-Mhp bivalent reference vaccines in an experimental Mhp challenge model. The main finding was that Vaccine A significantly reduced Mhp-associated macroscopic lung lesion scores compared with the unvaccinated challenged control group and had the numerically lowest mean score among the three evaluated vaccines. This conclusion is primarily supported by the macroscopic lung lesion scores, which are widely used as a key endpoint for assessing protection against Mhp-induced enzootic pneumonia [15,16]. A recent experimental study using the same commercial combination vaccine also reported less severe clinical signs and lung lesions following Mhp challenge [17].

The mean lung lesion score in the Vaccine A group was significantly lower than that in the unvaccinated challenged control group. In contrast, Vaccines B and C showed lower, non-significant reductions in lung lesion scores. Although the mean lung lesion score was numerically lower with Vaccine A, direct comparisons with Vaccines B and C were not statistically significant, and superiority over these vaccines was therefore not established.

The reduction in macroscopic lung lesions is clinically relevant because Mhp-associated lung damage can impair respiratory function and predispose pigs to secondary bacterial infections under field conditions [4]. Although no significant differences in average daily weight gain were observed in the present study, this may be related to the relatively short post-challenge observation period and the controlled experimental conditions. A previous field trial likewise reported significant differences in lung lesion severity between two Mhp vaccination strategies without a significant difference in average daily weight gain [18]. In commercial production systems, reduction in lung lesion severity may still have practical value by potentially improving respiratory health and reducing the impact of enzootic pneumonia over a longer production period [19].

The histopathological findings further supported the macroscopic lesion results. Severe peribronchiolar and perivascular lymphoid hyperplasia, inflammatory cell infiltration, and alveolar exudation were observed in the UnVac/Ch group, which are typical lesions associated with Mhp infection. In contrast, pigs in the Vaccine A group showed milder and more focal histopathological changes, and the overall lung architecture was largely preserved. Vaccines B and C showed more frequent and more severe histopathological lesions, which were more similar to those observed in the UnVac/Ch group. These observations suggest that Vaccine A not only reduced gross pulmonary lesions but also alleviated microscopic lung damage following Mhp challenge.

Pre-challenge Mhp-IgG seroconversion was detected in both the Vaccine A and Vaccine B groups by Day 21. The proportions of seropositive pigs were numerically higher in Vaccine A than in Vaccine B on Day 21 (10/18 vs. 6/18) and Day 35 (13/18 vs. 8/18). Vaccine C did not show detectable Mhp-IgG seroconversion before challenge. Notably, a previous field study detected Mhp-specific T-cell responses after commercial bacterin vaccination despite the absence of post-vaccination seroconversion [20]. Thus, the absence of detectable serum antibodies does not necessarily indicate the absence of a vaccine-induced immune response. One possible explanation is that the commercial ELISA may not detect antibody responses elicited by different vaccine antigen preparations equally well, although this possibility was not directly evaluated. Although the relationship between systemic Mhp-IgG and protection is complex, because Mhp mainly colonizes the respiratory mucosal surface, early seroconversion may still reflect efficient antigen recognition and immune activation after vaccination [21]. However, systemic IgG alone should not be interpreted as a complete correlate of protection against Mhp, and other immune mechanisms, including mucosal and cell-mediated responses, may also contribute to vaccine-induced protection.

Mhp DNA quantification in lung tissues provided additional information on pathogen presence after challenge. Mhp DNA was detected in all challenged groups, whereas no Mhp DNA was detected in the UnVac/UnCh group. Vaccine A had the numerically lowest Mhp DNA detection rate and mean DNA load among the three vaccinated groups. These numerical findings were consistent with the lower mean lung lesion score observed in this group; however, differences in Mhp DNA detection rates and DNA loads among the vaccinated groups were not statistically significant. Nevertheless, Mhp DNA remained detectable in a proportion of vaccinated pigs, indicating that vaccination reduced disease severity rather than completely preventing infection or colonization. This is consistent with the current understanding that Mhp bacterin-based vaccines generally provide partial protection, mainly by reducing clinical signs and lung lesions, rather than inducing sterilizing immunity [7,10,11].

All three vaccines were well tolerated, with no vaccine-related systemic or local adverse reactions observed during the post-vaccination monitoring period. In addition, all vaccinated groups developed PCV2-specific antibody responses, and 100% of vaccinated pigs were seropositive for PCV2 by Day 35. These results indicate that the PCV2 antigen components of the three bivalent vaccines were immunogenic under the conditions of this study. The unvaccinated control groups remained seronegative for PCV2 throughout the study, suggesting that no detectable PCV2 serological exposure occurred during the experimental period. However, because this study did not include a PCV2 challenge, the PCV2 serology results should be interpreted as evidence of antibody induction rather than direct evidence of PCV2 protective efficacy.

The numerical differences observed among the vaccinated groups may partly reflect differences in antigen composition or vaccine formulation. Experimental studies using different adjuvant formulations have demonstrated that formulation can influence protection against Mhp challenge [22]. However, this study did not directly investigate the genetic or antigenic relationship between vaccine components and the challenge strain. Therefore, the mechanisms underlying these numerical differences cannot be conclusively determined from the present data. A previous comparison of two commercial Mhp bacterins demonstrated differences in local and systemic immune responses and in protection against lung lesions, supporting the value of controlled comparative vaccine evaluations [23].

The absence of significant differences in ADWG among groups should also be interpreted in the context of the experimental design. The post-challenge observation period was limited to 28 days, and animals were maintained under controlled experimental conditions with limited environmental and microbial pressure. These conditions may not fully reproduce the chronic and cumulative impact of Mhp infection on growth performance in commercial production systems. Growth performance after PCV2-Mhp vaccination has also been evaluated under nursery field conditions [24]. Therefore, although lung lesion reduction was clearly demonstrated in the Vaccine A group, the potential long-term production benefits of this effect would require further evaluation under field conditions or in longer-term studies [19,25].

This study has several limitations. First, the experimental design focused on Mhp challenge, while PCV2 challenge was not performed. Therefore, the study primarily supports conclusions regarding protection against Mhp-associated pulmonary lesions, rather than comprehensive protection against both components of the bivalent vaccine. Second, the sample size may have limited the detection of differences in secondary outcomes, including coughing, ADWG, and pathogen load. Third, the 28-day post-challenge observation period may not fully capture the long-term effects of vaccination on productivity parameters across the growing-finishing period. Finally, the study did not include detailed analyses of mucosal immunity, cellular immune responses, or antigenic relatedness between the vaccine components and the challenge strain, which could help clarify the mechanisms underlying the observed differences in protection.

## 5. Conclusions

This experimental challenge study compared the efficacy of three commercial PCV2-Mhp bivalent vaccines under controlled Mhp challenge conditions. All three vaccines were well tolerated and induced PCV2-specific antibody responses; however, their protective efficacy against Mhp-associated pulmonary lesions differed among products.

Cirbloc^®^ M Hyo (Vaccine A) significantly reduced macroscopic lung lesion scores following experimental Mhp challenge compared with the unvaccinated challenged control group, with the numerically lowest mean score among the three evaluated vaccines. Vaccine A induced pre-challenge Mhp-specific IgG seroconversion, significantly reduced macroscopic lung lesion scores, alleviated histopathological changes, and showed the lowest Mhp DNA detection rate and a numerically lower Mhp DNA load in lung tissues.

Overall, these findings support the efficacy of Cirbloc^®^ M Hyo in reducing Mhp-induced lung pathology under experimental challenge conditions. Further field studies or dual-challenge studies would be valuable to assess its long-term production benefits and broader performance in PRDC control programs.

## Figures and Tables

**Figure 1:**
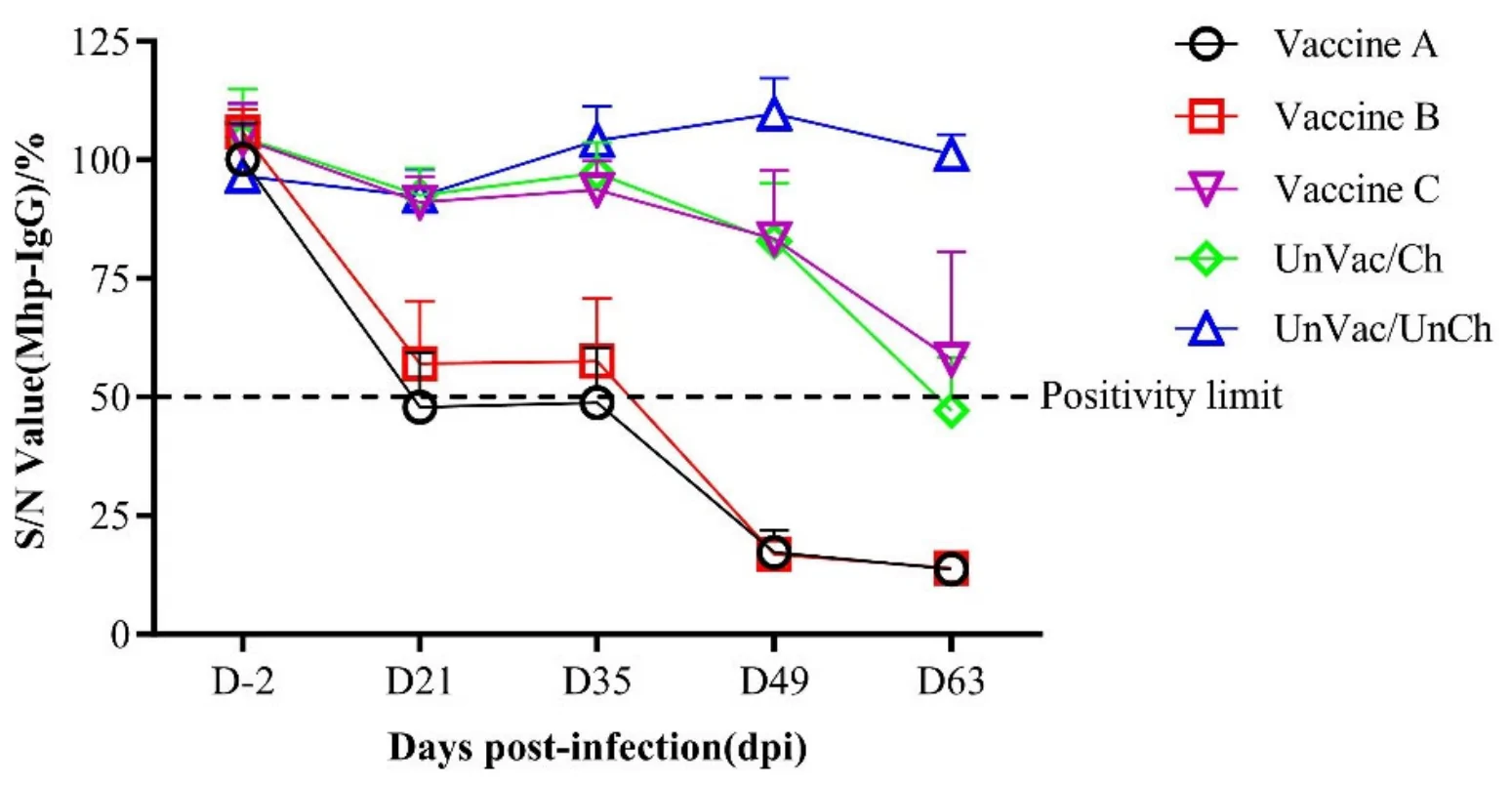
Dynamics of Mhp-specific IgG antibody responses in serum.

**Figure 2:**
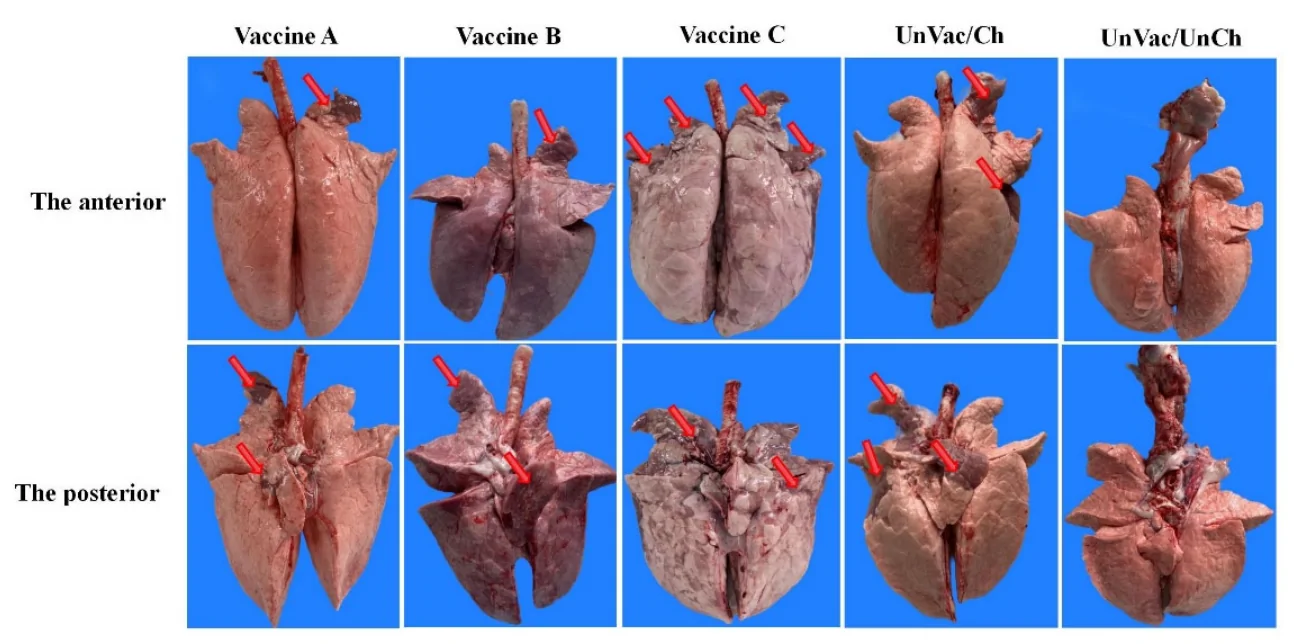
Representative macroscopic lung lesions in different groups after Mhp challenge. Red arrows indicate gross lung lesions compatible with enzootic pneumonia. (**Upper**) and (**lower**) panels show the anterior and posterior views of the lungs, respectively. Mhp, *Mycoplasma hyopneumoniae*; UnVac/Ch, unvaccinated challenged control group; UnVac/UnCh, unvaccinated unchallenged control group.

**Figure 3:**
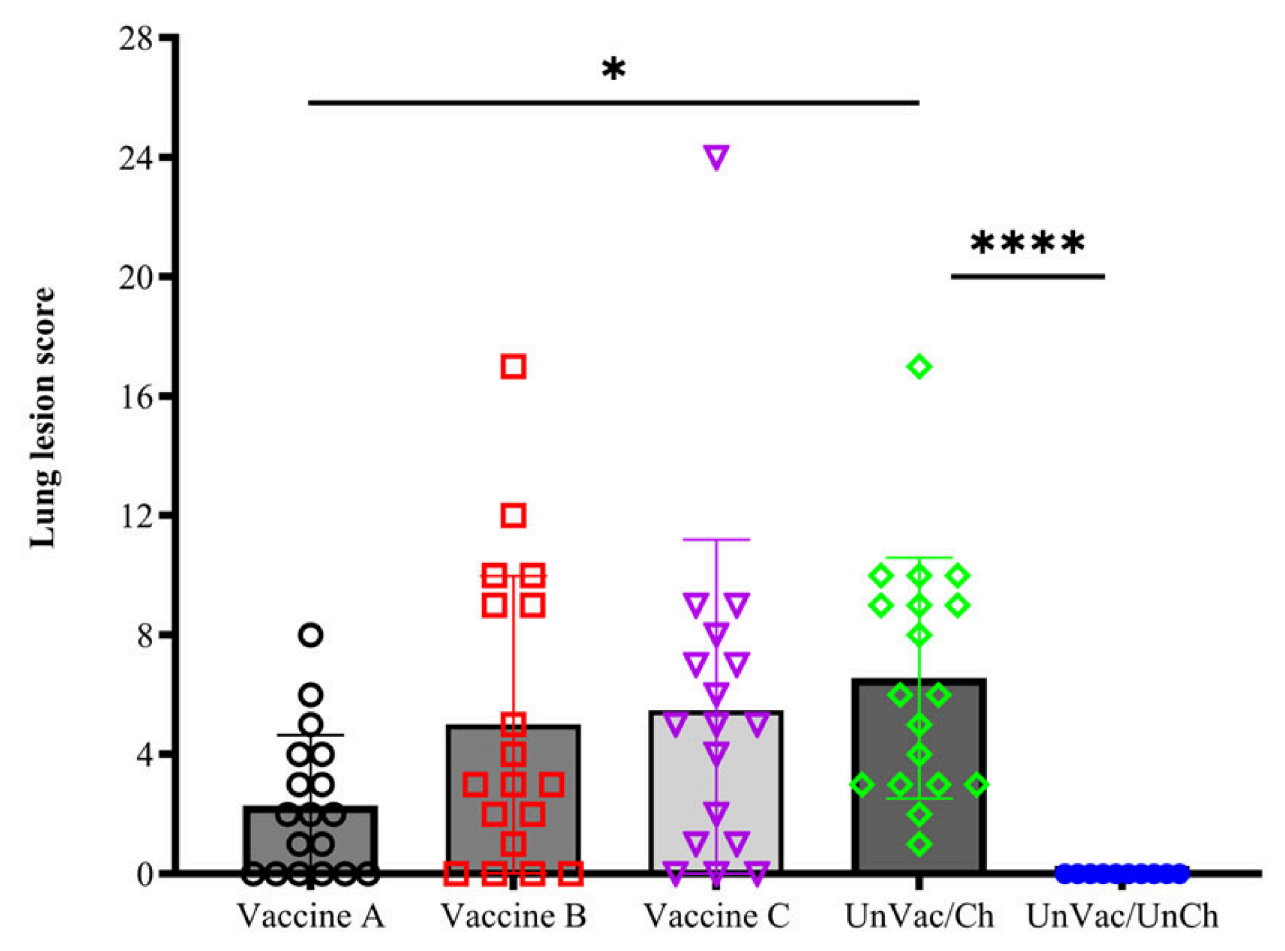
Individual macroscopic lung lesion scores in different groups. Columns and error bars indicate mean ± SD. Statistical comparisons were performed using the Kruskal–Wallis test followed by Dunn’s test with Bonferroni adjustment. * Adjusted *p* < 0.05; **** adjusted *p* < 0.0001. Symbols denote individual pigs: black circles, Vaccine A; red squares, Vaccine B; purple inverted triangles, Vaccine C; green diamonds, UnVac/Ch; blue circles, UnVac/UnCh.

**Figure 4:**
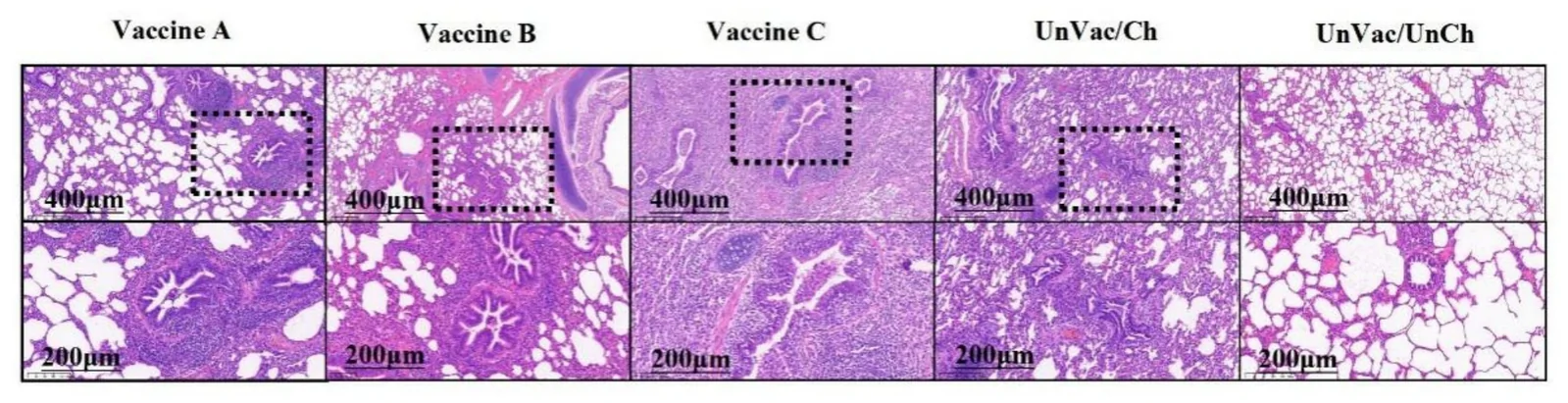
Representative histopathological findings in lung tissues from different groups. Lung sections were stained with hematoxylin and eosin (H & E). Scale bars are 400 μm in the (**upper**) panels and 200 μm in the (**lower**) panels. Where shown, dotted boxes in the upper panels mark regions displayed at higher magnification in the corresponding lower panels.

**Figure 5:**
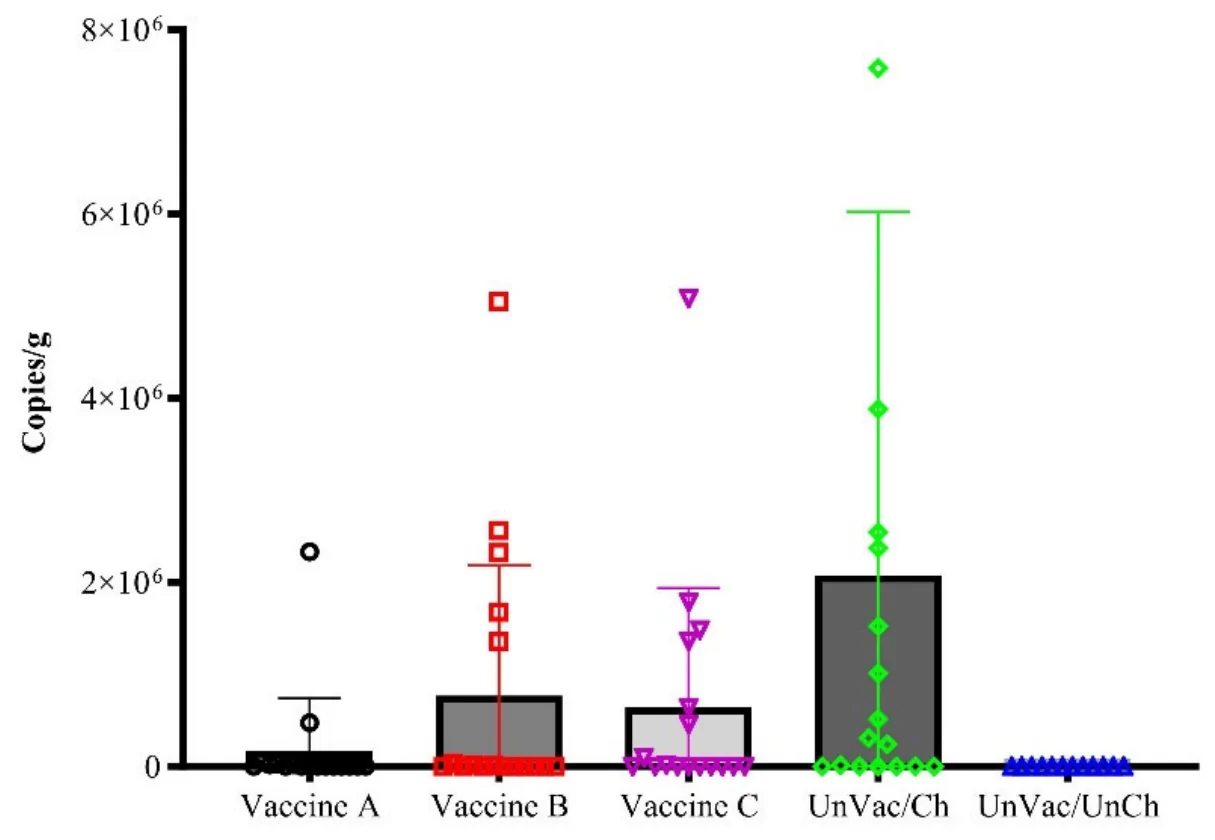
Mhp DNA loads in lung tissues quantified by qPCR. Results are expressed as copies per gram of lung tissue (copies/g). Bars and error bars indicate mean ± SD. Symbols denote individual pigs: black circles, Vaccine A; red squares, Vaccine B; purple inverted triangles, Vaccine C; green diamonds, UnVac/Ch; blue triangles, UnVac/UnCh.

**Table 1:** Design of the immunization experiment and details of the vaccines.

Group	Vaccine Antigen Types	Number of Piglets	Vaccination (3 WOA)	Challenge (8 WOA)	Necropsy (12 WOA)
Vaccine A	Recombinant baculovirus-expressed PCV2 Cap antigen + Inactivated Mhp antigen	18	Cirbloc^®^ M Hyo, 2.0 mL, IM	Mhp lung homogenate, 5 mL, IT	All
Vaccine B	Recombinant PCV2 antigen + inactivated Mhp antigen	18	2.0 mL, IM	Mhp lung homogenate, 5 mL, IT	All
Vaccine C	Recombinant PCV2 antigen + inactivated Mhp antigen	18	2.0 mL, IM	Mhp lung homogenate, 5 mL, IT	All
UnVac/Ch	PBS	18	2.0 mL, IM	Mhp lung homogenate, 5 mL, IT	All
UnVac/UnCh	PBS	10	2.0 mL, IM	—	All

Note: vaccines B and C are distinct commercial products. The entries in this column summarize antigen types and do not imply identical antigen composition or formulation. WOA: weeks of age; IM: intramuscular; IT: intratracheal; PBS: phosphate-buffered saline; Mhp: *Mycoplasma hyopneumoniae*.

**Table 2:** Coughing observations after Mhp challenge.

Group	D35–D42		D49		D56		D63	
	AM	PM	AM	PM	AM	PM	AM	PM
Vaccine A	0/18	0/18	0/18	0/18	1/18	0/18	1/18	0/18
Vaccine B	0/18	0/18	0/18	0/18	1/18	0/18	1/18	0/18
Vaccine C	0/17 *	0/17	0/17	0/17	0/17	0/17	1/17	0/17
UnVac/Ch	0/18	0/18	0/18	0/18	0/18	0/18	2/18	0/18
UnVac/UnCh	0/10	0/10	0/10	0/10	0/10	0/10	0/10	0/10

Note: data are presented as the number of pigs with coughing/the number of pigs observed at each time point. * One pig in the Vaccine C group died on Day 35 due to stress unrelated to vaccination or Mhp challenge, based on necropsy findings.

**Table 3:** Body weight and average daily weight gain of pigs in each group.

Group	Body Weight on Day-2 (kg)	Body Weight on Day 35 (kg)	Body Weight on Day 63 (kg)	ADWG from Day-2 to Day 63 (g/day)	ADWG from Day 35 to Day 63 (g/day)
Vaccine A	11.65 ± 2.42	22.82 ± 6.05	32.55 ± 8.52	326.30 ± 101.70	367.70 ± 103.90
Vaccine B	11.61 ± 2.18	22.07 ± 4.21	32.33 ± 4.74	318.80 ± 66.70	366.40 ± 126.80
Vaccine C	11.96 ± 1.70	23.37 ± 3.91	33.82 ± 5.18	336.40 ± 62.40	373.30 ± 79.00
UnVac/Ch	12.00 ± 2.67	22.40 ± 5.58	32.43 ± 6.98	314.30 ± 93.40	358.30 ± 150.10
UnVac/UnCh	12.10 ± 1.12	23.68 ± 1.92	33.81 ± 4.07	334.00 ± 64.00	362.00 ± 139.50

Note: data are expressed as mean ± SD. No significant differences were observed among groups at each time point or during each ADWG calculation period (*p* > 0.05).

**Table 4:** Mhp-specific IgG seroconversion rates in serum.

Group	D-2		D21		D35		D49		D63	
	Positive/Tested	Positive Rate (%)	Positive/Tested	Positive Rate (%)	Positive/Tested	Positive Rate (%)	Positive/Tested	Positive Rate (%)	Positive/Tested	Positive Rate (%)
Vaccine A	0/18	0	10/18	56	13/18	72	18/18	100	18/18	100
Vaccine B	0/18	0	6/18	33	8/18	44	18/18	100	18/18	100
Vaccine C	0/18	0	0/18	0	0/18	0	0/17	0	7/17	41
UnVac/Ch	0/18	0	0/18	0	0/18	0	0/18	0	7/18	39
UnVac/UnCh	0/10	0	0/10	0	0/10	0	0/10	0	0/10	0

Note: data are presented as the number of seropositive pigs/the number of pigs tested and the corresponding seropositivity rate.

**Table 5:** PCV2-specific antibody seropositivity rates in serum.

Group	D-2		D21		D35		D63	
	Positive Number/Tested	Positive Rate (%)	Positive Number/Tested	Positive Rate (%)	Positive Number/Tested	Positive Rate (%)	Positive Number/Tested	Positive Rate (%)
Vaccine A	0/18	0.00	13/18	72.22	18/18	100.00	18/18	100.00
Vaccine B	0/18	0.00	12/18	66.67	18/18	100.00	18/18	100.00
Vaccine C	0/18	0.00	11/18	61.11	18/18	100.00	17/17	100.00
UnVac/Ch	0/18	0.00	0/18	0.00	0/18	0.00	0/18	0.00
UnVac/UnCh	0/10	0.00	0/10	0.00	0/10	0.00	0/10	0.00

Note: data are presented as the number of seropositive pigs/the number of pigs tested and the corresponding seropositivity rate.

**Table 6:** Macroscopic lung lesion scores (Mean ± SD).

Group	Mean Lesion Score
Vaccine A	2.28 ± 2.37
Vaccine B	5.00 ± 4.98
Vaccine C	5.47 ± 5.72
UnVac/Ch	6.56 ± 4.03
UnVac/UnCh	0.00 ± 0.00

## Data Availability

The data presented in this study are available on request from the corresponding author.

## Abbreviations

The following abbreviations are used in this manuscript:

EP	Enzootic pneumonia
PRDC	Porcine respiratory disease complex
PCV2	Porcine circovirus type 2
Mhp	*Mycoplasma hyopneumoniae*
ADWG	Average daily weight gain
IM	Intramuscular
IT	Intratracheal
dpv	Days post-vaccination
wpc	Weeks post-challenge
SPF	Specific-pathogen-free
qPCR	Quantitative polymerase chain reaction
